# Hybrid ant colony-based inter-cluster routing protocol for FANET

**DOI:** 10.1038/s41598-024-64454-1

**Published:** 2024-07-07

**Authors:** Siwei Yang, Shu Wang, Tingli Li, Tao Hu, Ziliang Xu, Renze He, Bing Zhang

**Affiliations:** 1 School of Data and Target Engineering, PLA Strategic Support Force Information Engineering University, Zhengzhou, 450001 China; 2 27Th Research Institute of China Electronics Technology Group Corporation, Zhengzhou, 450001 China

**Keywords:** Mathematics and computing, Computer science, Information technology

## Abstract

This study addresses the challenges in large-scale unmanned aerial vehicle (UAV) clusters, specifically the scalability issues and limitations of using reactive routing protocols for inter-cluster routing. These traditional methods place an excessive burden on cluster heads and struggle to adapt to frequently changing topologies, leading to decreased network performance. To solve these problems, we propose an innovative inter-cluster routing protocol (ICRP), which is based on a hybrid ant colony algorithm. During the route establishment phase, ICRP uses this algorithm to identify the optimal relay node. This approach is inspired by the foraging behavior of Physarum polycephalum, combining factors such as the number of hops from the source node, the load condition of the node, and its weight in the pheromone calculation. In the route maintenance phase, ICRP uses a predictive repair and contraction mechanism to dynamically maintain routes, accommodating the high mobility of UAVs. Comparative simulations in OMNeT +  + showed that this protocol surpasses ad-hoc on-demand distance vector (AODV), fuzzy-logic-assisted-AODV, and Enhanced-Ant-AODV routing protocols in packet delivery rate and end-to-end transmission delay. Furthermore, it showed superior adaptation to network environments with high-speed node mobility.

## Introduction

In recent years, unmanned aerial vehicles (UAVs) have shown significant potential in both military and civilian applications. Military uses include reconnaissance^1^, target identification, and attack operations. In civilian context, UAVs are utilized for disaster monitoring^2^, agricultural plant protection^3^, and as aerial base stations^4^. However, the limitations of a single UAV in terms of energy, capability, and payload capacity make it unsuitable for complex missions. Therefore, multi-UAV cooperative operations have emerged as a solution, where rapid and reliable communication is a major challenge.

The concept of the flying ad-hoc network (FANET) has been introduced in response to this need. FANET, a dynamic formation network, introduces ideas from mobile and vehicle ad-hoc networks into UAV cooperative communication^5^. This concept enables UAVs to spontaneously form a communication network, facilitating information sharing with self-repair capabilities. FANET architectures are generally of two types: planar and hierarchical. In a planar structure, all nodes are at the same level and communicate directly, making it ideal for small networks. By contrast, large-scale UAV clusters often adopt a hierarchical structure, divided into sub-networks through clustering strategies for efficient, hierarchical management. Within these sub-networks, proactive routing protocols are preferred because of frequent node communication, necessitating quick route establishment. However, for inter-cluster communication, reactive routing protocols are mostly used in the backbone network, as demonstrated by existing research and protocols such as cluster based routing protocol (CBRP)^6^, which uses the dynamic source routing (DSR) protocol. Researchers^7–9^ have considered factors such as UAV residual energy and inter-UAV distances in creating multiple routes along the backbone network, selecting the most suitable for data forwarding. Despite their effectiveness, traditional reactive routing protocols face challenges and limitations when applied to cluster-structured networks with highly dynamic topologies.

Current research on reactive routing protocols primarily aims to enhance link stability during route discovery. The widespread use of flooding to propagate routing information often leads to network congestion and unstable links. To mitigate this issue, Mei et al.^10^ proposed a high-stability protocol that combines load prediction with a pseudo-gossip mechanism for balanced network load and reduced network overhead, thereby improving route establishment reliability. Chen et al.^11^ developed a method to calculate dynamic forwarding probability based on local neighboring nodes, incorporating cross-layer ideas such as node speed to determine link weights for route selection. Lee et al.^12^ suggested a novel approach where each node, during route initiation, assesses its suitability to forward a route request (RREQ) based on factors such as direction, residual energy, link quality, and stability. Typically, the destination node receives multiple RREQs, necessitating the selection of an optimal multi-hop route for the route reply (RREP) unlike the case in reported existing research. An et al.^13^ defined link retention probability to assess link connectivity stability. Similarly, Lee et al.^14^ proposed a routing mechanism focusing on path reliability and stability, evaluating links through duration and node congestion likelihood. The paths with high stability are identified by combining the two key metrics. Xufeng et al.^15^ introduced a link quality prediction-based on-demand routing algorithm, predicting link stability and node congestion using a gray-WNN model. This method aims to minimize the impact of UAV dynamics. Li et al.^16^ developed a fuzzy logic-assisted ad-hoc on-demand distance vector (AODV) routing algorithm that considers latency, stability, and residual energy, selecting the most reliable node for data transmission. Studies^17–21^ have introduced firefly and ant colony algorithms into routing protocols, optimizing node selection for the next hop based on factors such as energy consumption, buffer congestion, and hop count. To address the low fault tolerance in data transmission, an interference-resistant multipath routing protocol, considering link quality, traffic load, and spatial distance, has been proposed to identify optimal neighboring links^22,23^. For route maintenance, the fast repair of broken links is crucial. Qing et al.^24^ presented a protocol that selects the highest-energy node from common neighbors at a disconnection to re-establish the link, thus enhancing repair efficiency, reducing delay, and lowering routing overhead. In summary, while these reactive routing protocols offer various solutions for inter-cluster routing, they still present challenges in application, particularly in networks with highly dynamic topologies.

1. Excessive load on cluster-head nodes: in a cluster-structured network, cluster-head nodes often bear excessive computational and communication tasks. Reactive routing protocols typically fail to effectively distribute these tasks between the cluster-head nodes and other nodes, resulting in an imbalanced load.
2. Evaluation metrics are not comprehensive and efficient enough: current link stability metrics mainly focus on node movement, neglecting communication quality. Additionally, some metrics are computationally complex and inefficient, reducing their practicality in distributed network applications. Consequently, the computational and communication resource overhead increases, limiting scalability and efficiency.
3. Poor network fault tolerance: given the dynamic nature of node positions in inter-cluster routing, the network topology is prone to unpredictable changes, emphasizing the need for robust fault tolerance in routing algorithms. Reactive routing protocols often lack such fault-tolerant strategies, making them ill-equipped to handle topology changes.

To address these problems, this study proposes an inter-cluster routing protocol (ICRP), a novel protocol grounded in a hybrid ant colony algorithm and inspired by both ant colony behaviors and the foraging model of Physarum polycephalum. ICRP aims to reduce the load on cluster-head nodes by considering the weight of each node within the cluster network structure during the relay node selection process. This approach, coupled with a streamlined design of evaluation indices, improves the protocol’s adaptability in large-scale network scenarios. Furthermore, ICRP incorporates predictive repair and contraction mechanisms to improve its fault tolerance. These mechanisms account for potential link disconnections and bypasses, allowing for dynamic routing adjustments and efficient reduction in data transmission delays. In the route establishment phase, the protocol utilizes node pheromones and heuristic functions to select optimal relay nodes, thus constructing stable and efficient transmission links. During the route maintenance phase, ICRP effectively manages high UAV mobility and network topology changes, thereby improving transmission reliability. The innovative aspects of this algorithm, as presented in this paper, offer significant advancements compared to existing approaches.

1. In the route establishment phase, ICRP, during this phase, uses node pheromones and heuristic functions to select relay nodes. Pheromone concentration considers the number of hops from the source node, the node’s load, and its weight in the network structure. This approach aims to minimize the involvement of key nodes, such as cluster heads, in the route.
2. In the heuristic function design, inspired by the foraging behavior of Physarum polycephalum, to evaluate link communication, optimizing the selection of relay nodes and enhancing route stability.
3. In the route maintenance phase, a predictive repair and contraction mechanism is incorporated to dynamically adjust routing, accommodating high UAV mobility and network topology changes. This approach aims to reduce route disconnections and eliminate unnecessary relay nodes, thereby enhancing data transmission reliability.

The rest of the paper is organized as follows. In Section "Hybrid ant colony algorithm", we present an improved hybrid ant colony algorithm. In Section "Inter-cluster routing protocols", we provide a detailed explanation of the proposed ICRP. In Section "Simulation results and analysis", we compare and analyze the performance of the ICRP scheme with existing routing protocols, such as AODV, fuzzy-logic-assisted-AODV (FL-AODV), and Enhanced-Ant-AODV, through simulation experiments. Finally, in Section "Conclusion", we summarize our work and provide an overview of future research directions and potential work.

## Hybrid ant colony algorithm

The ant colony algorithm, as a heuristic search algorithm, demonstrates exceptional processing capacity and scalability through distributed computing when tackling complex or large-scale problems. Internally, it employs an active positive feedback mechanism, namely the accumulation of pheromones, to efficiently guide the ant colony in finding the optimal path, accelerating the optimization process. Simultaneously, it maintains an effective balance between exploring new paths and exploiting known paths, avoiding traps of local optima and enhancing its global search capability. This algorithm exhibits strong robustness and adaptability, capable of self-adjusting according to environmental changes and highly tolerant of abnormal behavior. Owing to these advantages, an increasing number of researchers are incorporating it into the design and optimization of routing protocols^25^. Despite its strengths, the ant colony optimization (ACO) algorithm faces shortcomings. For example, the pheromone updating mechanism might lead to a slower convergence speed, especially during the initial iterations. This is because the uneven distribution of pheromones might require several rounds of iterations before significantly guiding the search direction. Furthermore, in dynamically changing networks, previously optimal paths may no longer be viable due to environmental changes. The ant colony algorithm needs time to re-accumulate pheromones to adapt to the new optimal paths, affecting its ability to cope with rapidly changing environments. To overcome these limitations, this study proposes optimizations to the pheromone updating rule and introduces the foraging behavior model of Physarum polycephalum as a heuristic function. This function considers the stability of the links, remaining energy of the nodes, and quality of link communication. By optimizing the path selection process, it not only enhances the adaptability and energy use efficiency of the network but also improves the network stability and communication quality.

### Pheromone update rules

In the natural world, ants use pheromone trails as a communication tool to coordinate and achieve efficient foraging. Similarly, in the ant colony algorithm, pheromones are important for guiding the search and optimization process, aiding in the swift identification of optimal solutions. The pheromone update model presented in this paper is structured as follows:

1 $${\tau }_{i}(t+\Delta t)=(1-\rho ){\tau }_{i}(t)+\rho \times\Delta {\tau }_{i}(t){\tau }_{i}(t+\Delta t)=(1-\rho ){\tau }_{i}(t)+\rho \times\Delta {\tau }_{i}(t)$$

2 $$\Delta {\tau }_{i}(t)=({q}_{f}/{q}_{t})/(ho{p}_{i}\times impor{t}_{i})$$

where $\Delta {\tau }_{i}(t)$, ${\tau }_{i}(t+\Delta t)$ denote the pheromone in the message and updated value of the message pheromone, respectively, $\rho$ represents the pheromone volatilization coefficient ($\rho \in (\text{0,1})$), which is used to constraint the increase of pheromone. ${q}_{f}$ denotes the idle queue length of the receiving queue of node $i$, ${q}_{t}$ stands for the total length of the receiving queue of node $i$,$ho{p}_{i}$ signifies the number of routing hops from the source node to node $i$, and $impor{t}_{i}$ denotes the weight of the node $i$. The more critical the node's position is in the structure of the clustered network, the greater the weight is.

The pheromone update rules consider the node load, the number of hops to the source node, and node weights. Considering node load, the algorithm can avoid overly relying on nodes that may already be overloaded, improving the overall quality of the path and the efficiency of network resource use. Avoiding overloaded nodes helps reduce congestion, potentially increasing the throughput of the path and reduce latency. Considering the number of hops to the source node helps minimize the path length and communication delay. This method optimizes the transmission time of data in the network, making the path as short and efficient as possible. Fewer hops mean that information can be conveyed faster, which is important for network services that require quick responses, such as real-time data processing and sensitive communication systems. Considering node weights and decreasing them in the order of cluster heads, deputy cluster heads, and ordinary cluster members, reduce the number of critical nodes in the transmission path, thereby lowering the risk of critical node overload and reducing network service interruptions caused by overload of critical nodes.

### Heuristic function design

Physarum polycephalum, a unicellular fungus, demonstrates remarkable adaptability in complex conditions by optimizing resource allocation and information dissemination through a self-organized network of hyphae. Similarly, FANET, a self-organized network of UAVs, operates under complex conditions and environmental constraints, necessitating comparable self-adaptation and information transmission capabilities. Utilizing the foraging model of Physarum polycephalum as the heuristic function in the ACO algorithm can significantly enhance its adaptability and search capability. The foraging model is described as follows:

3 $${Q}_{ij}=\frac{\pi {r}_{ij}^{4}\left({P}_{i}-{P}_{j}\right)}{8\eta {L}_{ij}}=\frac{{D}_{ij}\left({P}_{i}-{P}_{j}\right)}{{L}_{ij}}=\frac{{D}_{ij}\Delta {P}_{ij}}{{L}_{ij}}$$

where ${Q}_{ij}$ denotes the fluid flux per unit time through a tubular body $ij$, ${D}_{ij}=\pi {r}_{ij}^{4}/8\eta$ represents the flowability of the tubule, ${r}_{ij}$ stands for the radius of the tubule, and $\eta$ denotes fluid viscosity inside the tubule. $\Delta {P}_{ij}={P}_{i}-{P}_{j}$ indicates the pressure difference between the fluid at the ends of the tubule, and ${L}_{ij}$ signifies the length of the tubule.

We employ the dimensionless analysis method to map the Physarum mathematical model to the FANET. ${D}_{ij}$ represents an intrinsic property of the tubular body, which denotes the physical parameter for the tubular body's fluid transport capacity. Therefore, we substitute ${D}_{ij}$ with bandwidth ${B}_{ij}$ to represent the data transmission capacity of the link between node $i$ and node $j$. Considering path loss in FANET, where greater distances between nodes result in higher loss, the path loss between nodes ${L}_{ij}^{\alpha }$ is used instead of the length of the tubular ${L}_{ij}$.

The magnitude of the pressure difference $\Delta {P}_{ij}$ of the tubular body determines the strength of the fluid flow. The greater the pressure difference, the stronger the fluid's transport capacity. Similarly, in FANET, a reliable communication link is crucial for achieving efficient data transmission. Unstable communication links and lower residual energy can lead to unstable connection status, which may interrupt communication. Additionally, poor link communication quality increases the likelihood of communication interference, leading to data transmission errors, delays, or interruptions. Therefore, in FANET, $\Delta {P}_{ij}$ can be expressed as follows:

4 $$\Delta {P}_{ij}={k}_{1}\times L{S}_{ij}+{k}_{2}\times E{R}_{ij}+{k}_{3}\times L{Q}_{ij}$$

where $L{S}_{ij}$, $E{R}_{ij}$ and $L{Q}_{ij}$ denote the link stability, minimum residual energy and link communication quality between two neighboring nodes, respectively. The weighting factors ${k}_{1}$,${k}_{2}$, ${k}_{3}$ each contribute to these variables, with their sum equaling 1.

In the model representing the foraging by Physarum polycephalum, ${Q}_{ij}$ represents the fluid flux per unit time through a tube $ij$, and in the FANET, ${Q}_{ij}$ denotes the virtual traffic that can pass through the link $ij$. This leads to a modification of Eq. (3) for its application in the network context:

5 $${Q}_{ij}=\frac{{B}_{ij}\Delta {P}_{ij}}{{L}_{ij}^{\alpha }}=\frac{{B}_{ij}}{{L}_{ij}^{\alpha }}\left({k}_{1}\times L{S}_{ij}+{k}_{2}\times E{R}_{ij}+{k}_{3}\times L{Q}_{ij}\right)$$

## Inter-cluster routing protocols

**Figure 1:**
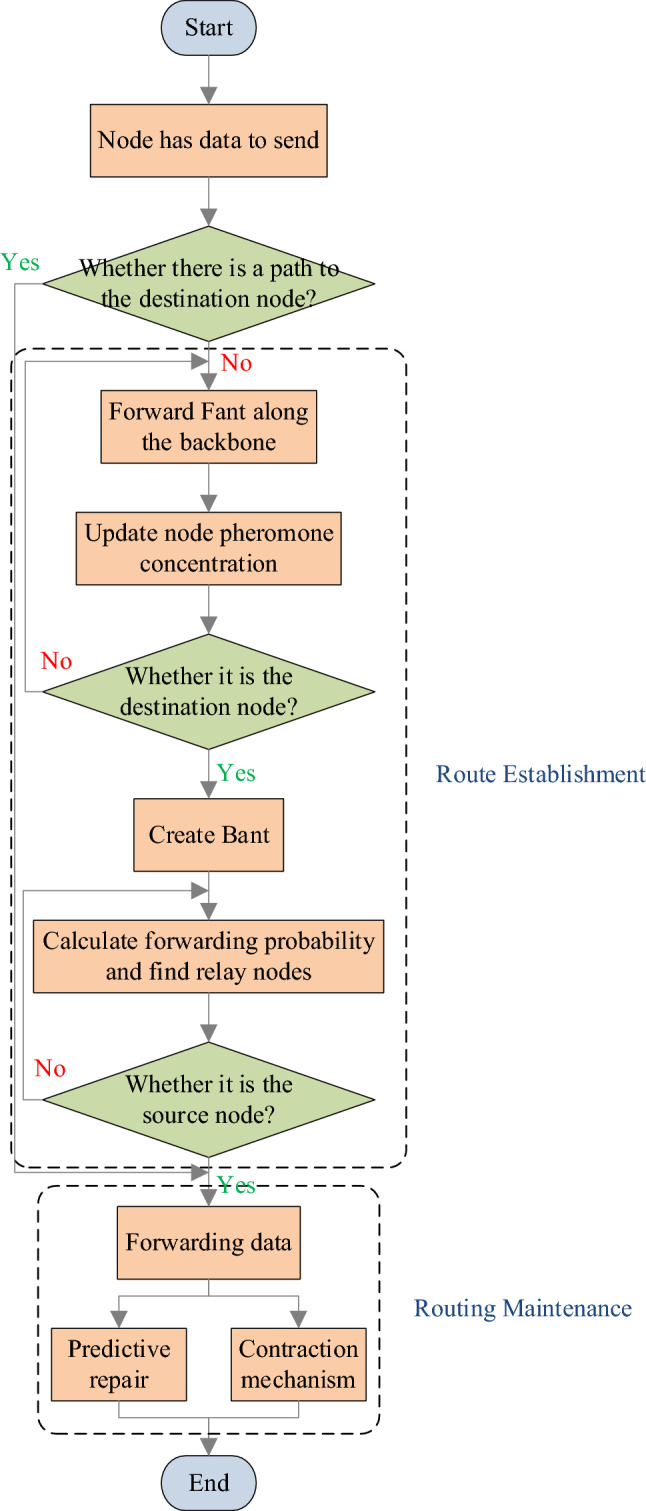
ICRP flowchart.

This section outlines the proposed ICRP and includes a flowchart, as shown in Fig. 1. The protocol is divided into two main phases: route establishment and route maintenance. At the stage of route establishment, forward ants search for destination nodes through the backbone network and update the pheromone concentration of nodes along the route to provide a basis for route selection. When the destination node is reached, reverse ants are sent, and the relay node is selected based on the forwarding probability of each node to establish a path from the destination node to the source node. In the route maintenance phase, the route path is updated and maintained in real time by predicting the link between nodes that will be disconnected and adopting the shrinking strategy. In this process, the deployment of forward and reverse ants significantly optimizes route construction, while maintenance mechanisms ensure that established routes can adapt to dynamic changes in the network environment. Table 1 provides a glossary of the symbols used.

**Table 1:** Relevant definitions.

Notation	Concrete meaning
HELLO	Beacon packet
Fant	Forward ant
Bant	Backward ant
Pheromone	Node pheromone
Rant	Repair ant
Sant	Shrink ant
SantACK	Shrink reply
${N}_{table}$	Neighbor table

### Route establishment

Traditional routing protocols employing the ant colony algorithm usually update node pheromones during the forwarding of RREPs along the reverse route, influencing the subsequent route establishment. However, this method can be problematic, as it relies on pheromone information based on previous path selections and reverse forwarding, which may not accurately reflect the current network state. To enhance accuracy and efficiency, each network node periodically sends a HELLO message to ascertain the neighboring nodes' pheromone levels and calculate virtual traffic. When a source node needs to send data, it first checks its routing table for a valid path to the destination. If no such path exists, it broadcasts a Fant message. An intermediate node receiving the Fant message for the first time updates its hop count to the source node and adjusts its pheromone concentration accordingly. If the node is part of the backbone network, it modifies the hop count in the Fant message, records its pheromone level in the message, and forwards it immediately. Otherwise, the message is discarded. The destination node, after receiving the Fant message, opens a time window to tally incoming Fant messages. Upon reaching a preset number or at the end of the time window, the collection process ceases. The destination node then calculates the probability of each neighboring node being the next hop and sends a Bant message to the most probable node. Intermediate nodes receiving the Bant message mark themselves as relay nodes, establish forward routes in their routing tables, and repeat the process until the Bant message reaches the source node, thereby establishing the route.

As per the previous section, the probability of node $i$ selecting node $j$ as the next hop at time *t* is denoted as ${P}_{ij}(t)$, :

6 $${P}_{ij}(t)=\left\{\begin{array}{c}\frac{{\left[{\tau }_{ij}(t)\right]}^{\alpha }\times {\left[{Q}_{ij}(t)\right]}^{\beta }}{{\sum }_{s\in {N}_{tabl{e}_{i}}}{\left[{\tau }_{is}(t)\right]}^{\alpha }\times {\left[{Q}_{is}(t)\right]}^{\beta }},j\in {N}_{tabl{e}_{i}}\\ 0,j\notin {N}_{tabl{e}_{i}}\end{array}\right.$$

where $\alpha$ is the pheromone factor that indicates the importance of pheromone quantity in the next-hop selection process, while $\beta$ is the heuristic function factor, which indicates the importance of the heuristic function value in this decision-making process.

Equation (5) indicates that virtual traffic ${Q}_{ij}(t)$ is influenced by link stability $L{S}_{ij}$, node residual energy $E{R}_{ij}$ and link communication quality $L{Q}_{ij}$.

(1) Link stability.

In the context of the free space fading model, the received power ${P}_{r}$ at a distance $d$ from the transmitting node can be expressed as

7 $${P}_{r}=\frac{{P}_{t}{G}_{t}{G}_{r}{\lambda }^{2}}{(4\pi {)}^{2}{d}^{2}L},$$

where ${P}_{t}$ signifies the transmit power of the antenna, ${G}_{t}$ denotes the gain of the transmitting antenna, ${G}_{r}$ denotes the gain of the receiving antenna, $\lambda$ represents the wavelength of the radio wave, and $L$ is the system loss factor.

Assuming that the antenna coverage radius is $R$, we define the threshold value for receiving HELLO messages from neighboring nodes as

8 $${P}_{threshold }=\frac{{P}_{t}{G}_{t}{G}_{r}{\lambda }^{2}}{(4\pi {)}^{2}{R}^{2}L}.$$

Based on the actual measured received power and threshold value of the received power, we define the variable ${X}_{ij}$ as the ratio of these two values.

9 $${X}_{ij}=1-\frac{{P}_{threshold }}{{P}_{r}}$$

Assuming that the expectation of ${X}_{ij}$ is $E\left({X}_{ij}\right)$ and variance is $D\left({X}_{ij}\right)$, according to the Anneme–Chebyshev inequality $P\left\{\left|{X}_{ij}-E\left({X}_{ij}\right)\right|<\varepsilon \right\}\ge 1-\frac{D\left({X}_{ij}\right)}{{\varepsilon }^{2}}$, if the variance approaches 0, it implies that the closer variable ${X}_{ij}$ is to its expectation, indicating less variability in ${X}_{ij}$. The variance of variable ${X}_{ij}$ is calculated as

10 $$D\left({X}_{ij}\right)=\left({\sum }_{n}\frac{{X}_{ij}^{2}}{n}\right)-{\left({\sum }_{n}\frac{{X}_{ij}}{n}\right)}^{2}$$

Based on the variance, we can infer the movement of neighboring node $j$ relative to the current node $i$. Therefore, the stability of motion between nodes can be described as

11 $$L{S}_{ij}=1-D\left({X}_{ij}\right).$$

In a certain period of time, when the movement between neighboring nodes is more frequent or unstable, it will cause significant fluctuations in signal receiving power, resulting in increased changes in ${X}_{ij}$, which increases the value of $D\left({X}_{ij}\right)$, and ultimately leads to a decrease in the value of $L{S}_{ij}$ and vice versa.

(2) Node residual energy.

The residual energy is usually derived from the initial energy of the UAV minus the total energy consumed since the start of the operation. In this study, the initial energy of each node (${E}_{I}$) is the same, and the total energy consumption (${E}_{T}$) includes communication between UAVs (${E}_{c}$), flight operations (${E}_{f}$), and sensor operation (${E}_{s}$), as per the following equations.

12 $${E}_{T}={E}_{c}+{E}_{f}+{E}_{s},$$

13 $${E}_{c}={E}_{Tx}+{E}_{Rx},$$

14 $${E}_{Tx} ={E}_{elec}\times l+{\varepsilon }_{fs}\times l\times {d}^{2},$$

15 $${E}_{Rx} ={E}_{elec}\times l,$$

where ${E}_{Tx}$ denotes the energy used to send a signal, ${E}_{Rx}$ signifies the energy used to receive a signal, ${E}_{elec}$ indicates the energy consumed per bit of data transmission, $l$ stands for the number of bits of the transmitted data, ${\varepsilon }_{fs}$ signifies the energy consumption coefficient of the amplifier, and $d$ represents the distance between the transmitter and receiver.

Then, the residual energy of each node (${E}_{R}$) can be calculated as

16 $${E}_{R}={E}_{I}-{E}_{T},$$

Therefore, $E{R}_{ij}$ can be denoted as

17 $$E{R}_{ij}=\mathit{min}({E}_{Ri},{E}_{Rj})/{E}_{I}.$$

(3) Link communication quality.

The packet reception rate ($PRR$) is often used as a soft metric to assess the quality of wireless link communications; however, the method is time-consuming and does not provide immediate results. In this study, we estimate $PRR$ from the received signal strength $RSSI$ following the approach in^26^.

The $BER$ for BPSK is expressed using the $Q$ function:

18 $$BER=Q\left(\sqrt{2SNR}\right).$$

The packet acceptance rate is

19 $$PRR=[1-Q\left(\sqrt{2SNR}\right){]}^{Nbit},$$

where $Nbit$ is the number of bits in the packet. The power of the received signal, is typically available at $RSSI$, and it relates to $SNR$ as follows:

20 $$SNR=\frac{{P}_{RSSI}-{P}_{N}}{{P}_{N}}=\frac{{P}_{RSSI}}{{P}_{N}}-1=1{0}^{\frac{RSSI-{N}_{dBm}}{10}}-1,$$

where ${P}_{RSSI}$ and ${P}_{N}$ are the received signal and background noise powers in mW, respectively, and $RSSI$ and ${N}_{dBm}$ are the measured values in dBm corresponding to ${P}_{RSSI}$ and ${P}_{N}$, respectively. Therefore, the relationship between $PRR$ and $RSSI$ can be expressed as follows:

21 $$PRR={\left[1-Q\left(\sqrt{2\times \left(1{0}^{\frac{RSSI-{N}_{dBm}}{10}}-1\right)}\right)\right]}^{Nbit}.$$

The $Q$ function can be simplified as

22 $$Q\left(x\right)=\frac{1}{2}\mathit{erfc}\left(\frac{1}{\sqrt{2}}x\right)=\frac{1}{2}-\frac{1}{2}\mathit{erf}\left(\frac{1}{\sqrt{2}}x\right),$$

23 $$erf\left(x\right)\approx \sqrt{1-{e}^{a{x}^{2}}},$$

where $a\in [-2,-1]$,$and L{Q}_{ij}$ is expressed as

24 $$L{Q}_{ij}={\left[\frac{1}{2}+\frac{1}{2}\times \sqrt{1-{e}^{a\times \left(1{0}^{\frac{RSSI-{N}_{dBm}}{10}}-1\right)}}\right]}^{Nbit}.$$

### Route maintenance

Fault tolerance technology is introduced in previous studies to enhance the robustness and self-healing ability of wireless networks^27^. Especially in the dynamic networking scenario of FANET, it is particularly important to update and maintain routing information in time because of the highly dynamic operating environment. This not only helps to quickly adapt to network topology changes caused by drone movement or failure, ensuring continuity and efficiency of data transmission but also significantly reduces transmission delays and saves energy by selecting the optimal transmission path. In addition, the optimized routing information maintenance enhances the scalability and flexibility of the network, improves the robustness of the network against failures, and supports advanced network functions, such as multipath routing, ensuring the performance and stability of the UAV network, which is critical for maintaining the highest performance of the network. When a path is established, each relay node $i$ monitors the virtual traffic to its next-hop node $j$ through periodically sent HELLO messages. When ${Q}_{ij}<{Q}_{min}$ between two points, node $i$ initiates a search for cooperative nodes by sending a Rant message. Upon receiving the Rant message, surrounding nodes assess whether they can establish a forward route to node $j$. If they can, they send an acknowledgment message back to node $i$, which then selects the most suitable node from the received acknowledgments as the cooperative node and updates its next-hop path accordingly.

**Figure 2:**
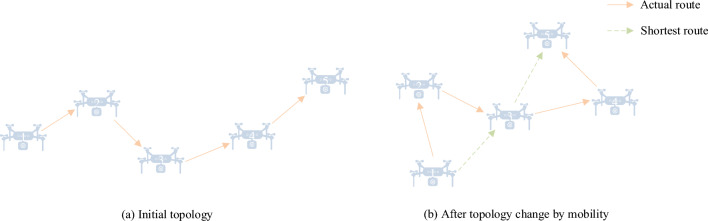
Hop-count reduction problem in the same route.

In the dynamic network environment, although the predictive repair mechanism can reduce the probability of route interruption, it may cause the increase of route hops and detouring, as shown in Fig. 2. Therefore, the shrink mechanism is introduced to eliminate unnecessary relay nodes in the routing to shorten the path. If the source node has a packet to send and a relevant route exists, it broadcasts a Sant message along the route with a certain probability. Upon receiving the Sant message for the first time, a node updates the hop count from itself to the source node. If it is the intended recipient of the packet, it also broadcasts the Sant message to the next-hop node and sends a SantACK acknowledgment to inform the previous hop node that it is not on the shortest route. Furthermore, it sends a SantACK acknowledgment message to inform itself that it can be the next-hop node. If the node receives a Sant for a second time and it is the intended recipient, it checks which node sent the first Sant, sends a SantACK acknowledgment to that node, and then forwards the Sant to the next hop. This mechanism can effectively adjust the routing path and quickly adapt to the high dynamic movement of the UAV, thereby shortening the path length, improving the adaptation speed of the network topology, and reducing the transmission delay and the risk of path failure. Shorter routing paths not only help save bandwidth and energy and reduce network congestion but also enhance network stability and throughput. In addition, the route shrinking mechanism also significantly improves the scalability and performance of large-scale FANET, making it more suitable for application scenarios with strict requirements for high efficiency and high reliability, effectively promoting the optimal utilization of resources and ensuring the continuous and efficient operation of the network.

## Simulation results and analysis

In this study, we simulate ICRP using OMNeT +  + network simulator and compare ICRP with AODV, fuzzy-logic-assisted-AODV^13^, and Enhanced-Ant-AODV^14^ routing protocols by changing the simulation parameters. For the comparison, we adopted the approach of existing inter-cluster routing protocols and modified the three comparison protocols based on the CBRP protocol, restricting the forwarding range of route request messages to the backbone network.

We chose OMNeT +  + as our simulator primarily due to its highly flexible framework, which enables the easy customization of complex network simulation environments. This simulator exhibits remarkable efficiency in handling large volumes of events and, through extensive libraries such as INET, supports a wide range of network protocols and device simulations, providing a vast platform for research work. Furthermore, OMNeT +  + boasts powerful visualization tools like Qtenv and Tkenv, which significantly enhance the intuitiveness and accessibility of the simulation process and result analysis. Given the diverse requirements of different networking scenarios in terms of node mobility speed and density, we have referenced document^28^ in setting our experimental simulation parameters. The specific simulation parameters are shown in Table 2.

**Table 2:** Parameter settings for simulation experiments.

Simulation parameter	Parameter value
Simulation time	120 s
Simulation scene size	1500 × 1500 m^2^
Number of nodes	40, 60, 80, 100, 120, 140
Maximum speed	20–60 m/s
Communication distance	200 m
Moving model	Gauss-Markov movement model
MAC protocol	IEEE802.11
Carrier frequency	2.4 GHz
Data group size	125 bytes
Data stream	CBR 100 kbps
Starting energy	5 J

### Average end-to-end delay

**Figure 3:**
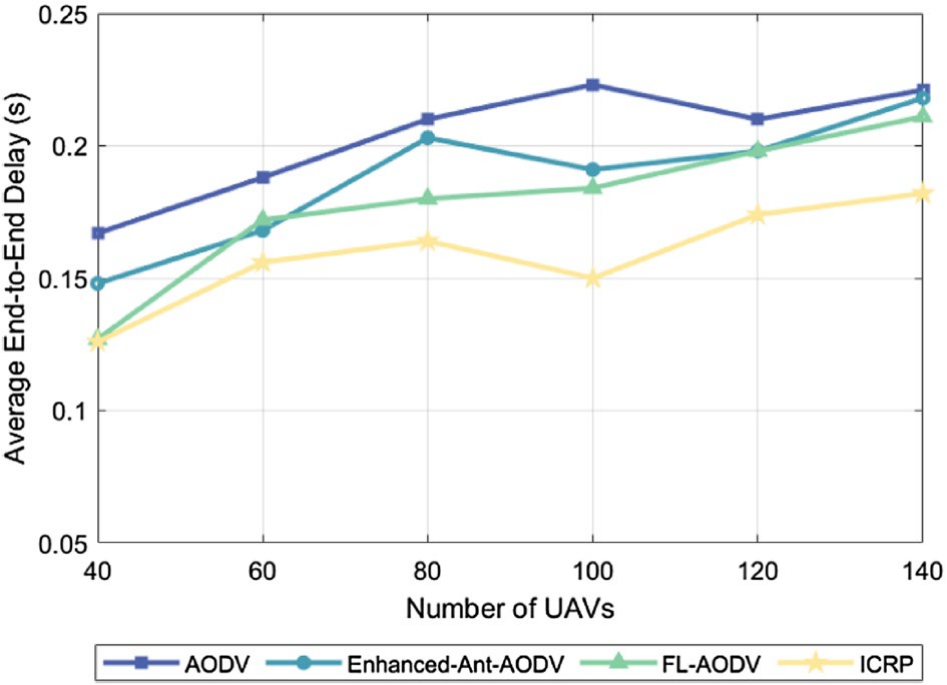
Performance comparison of average end-to-end delay for different number of nodes.

**Figure 4:**
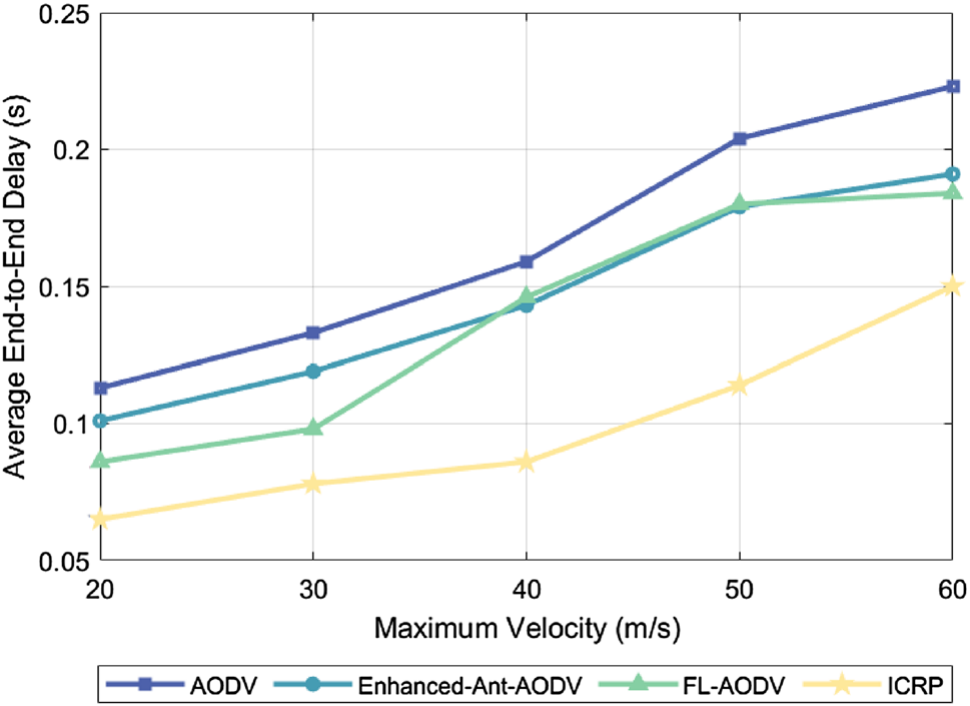
Performance comparison of average end-to-end delay for different maximum node speeds.

The average end-to-end delay is the average time it takes for a packet to travel from the source node to the destination node. This index can directly reflect the transmission efficiency of the network and the response speed of the routing protocol, which is critical for application scenarios with high real-time requirements. This is because an increase in the number of nodes or their mobility speed causes frequent changes in the network topology, leading to an increase in the latency of route discovery and maintenance. The nodes take more time to find the available routing paths, which increases the latency of data transmission. In addition, the increased number of nodes and mobility speed increases the possibility of packet conflicts, leading to packet loss and retransmission, further extending the transmission delay. Efficient routing protocols that establish more stable transmission links enable nodes to communicate more rapidly and consistently, thereby reducing the end-to-end delay. Figure 3 shows the comparison of average end-to-end delay for the AODV, Enhanced-Ant-AODV, FL-AODV, and ICRP protocols across various node counts at a maximum flight speed of 60 m/s. The ICRP routing protocol, as proposed in this paper, demonstrated the lowest average end-to-end delay, showing a reduction of 21.83%, 15.22%, and 10.55% respectively, compared to the other three protocols. Figure 4 shows a comparison of the average end-to-end delay of these four routing protocols for 100 UAVs at varying maximum flight speeds. ICRP showed a significant reduction in average end-to-end delay by 41.32%, 33.55%, and 28.21% compared to the other three routing protocols. The above two figures prove the excellent performance of ICRP in path optimization, network scalability, robustness, and resource management. This enhanced performance is attributed to the fact that ICRP attempts to choose non-cluster head nodes as relay nodes when establishing reverse routes. Additionally, during the route maintenance phase, ICRP dynamically adjusts the established routes to accommodate changes in the network topology. This strategy helps to reduce packet retransmissions and queue waiting time, thereby decreasing end-to-end latency. In contrast, routing protocols such as AODV, Enhanced-Ant-AODV, and FL-AODV establish routes along the backbone network, which increases the load pressure on the cluster-head nodes and leads to an increase in queuing time, which increases the average end-to-end delay.

### Package delivery rate

**Figure 5:**
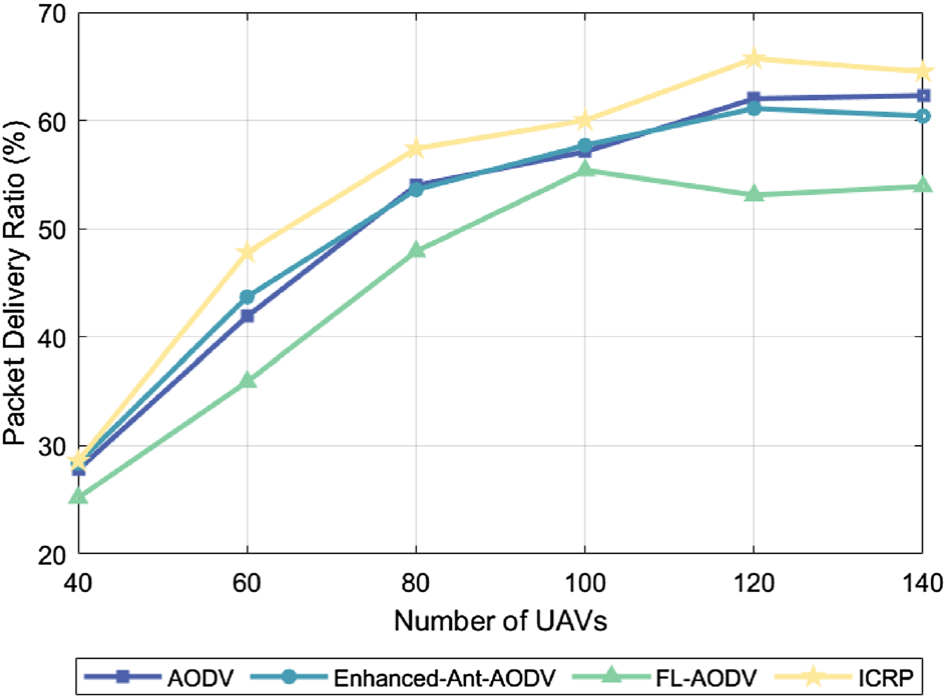
Performance comparison of packet delivery rate with different number of nodes.

**Figure 6:**
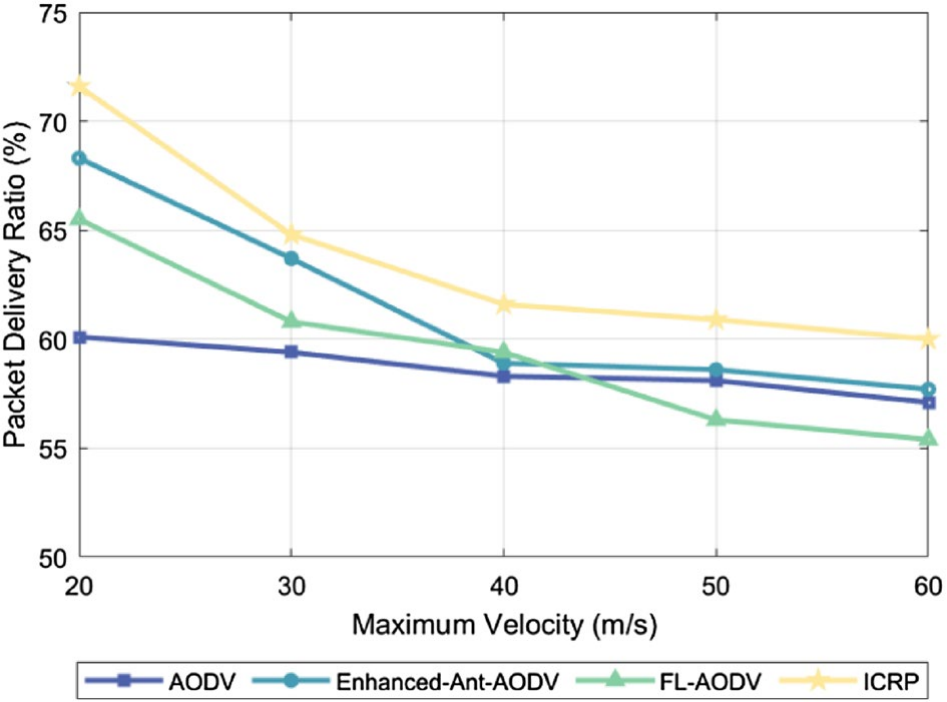
Performance comparison of packet delivery rate at different maximum node speeds.

The packet delivery ratio is an important metric in network performance, indicating the percentage of successfully transmitted packets. It represents the percentage of the total number of packets received by the destination node to the total number of packets sent by the source node. A high packet delivery rate indicates that the routing protocol can effectively adapt to the dynamic changes of the network, such as node movement, link interruption, or network congestion, and ensure the stable transmission of data in complex environments. Notably, the packet delivery rate tends to increase as the number of UAVs increases and decrease as UAV flight speed increases. This trend occurs because the connectivity between network nodes increases as the cluster density increases, providing more potential paths for packet transmission. This means that once a communication link fails, the system can quickly find an alternative path, increasing network redundancy, effectively improving the probability of successful packet delivery. However, network topology changes more frequently as the UAV flight speed increases, posing new challenges to the establishment and maintenance of communication links. High-speed moving drones may cause links to break down more frequently, increasing the risk that packets will be lost in transit, and as a result, overall packet delivery rates may decrease. A higher packet delivery rate means that an algorithm is more adaptable and robust against dynamic link changes. Figure 5 compares the packet delivery rates of the AODV, Enhanced-Ant-AODV, FL-AODV, and ICRP protocols across varying numbers of UAVs. The proposed ICRP routing protocol had the highest average packet delivery rate, showing improvements of 6.31%, 5.91%, and 19.7%, respectively, over AODV, Enhanced-Ant-AODV, and FL-AODV. Figure 6 shows the packet delivery rates of these protocols versus the maximum flight speed of UAVs. The packet delivery rate of ICRP improved by 8.76%, 3.81%, and 7.21%, respectively, compared to AODV, Enhanced-Ant-AODV, and FL-AODV. The superior performance of the ICRP protocol can be attributed to its adoption of predictive repair and contraction mechanisms, which effectively reduces the probability of link disconnection and thereby improves the success rate of packet deliveries. By contrast, the AODV, Enhanced-Ant-AODV, and FL-AODV routing protocols pay less attention to route maintenance, potentially leading to more frequent link disconnections and bypass issues, and consequently, higher packet loss rates.

### Energy consumption

**Figure 7:**
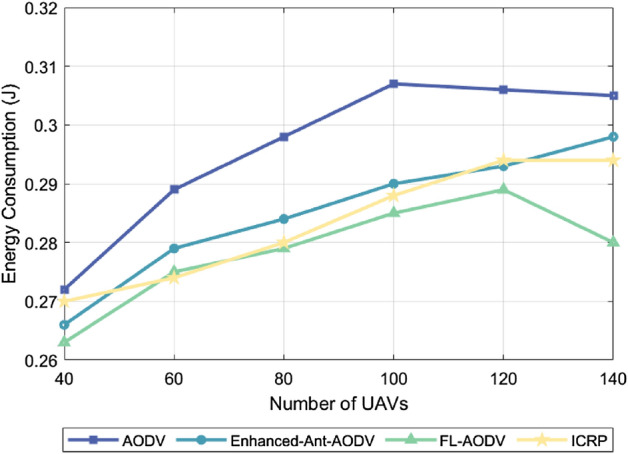
Performance comparison of energy consumption with different number of nodes.

**Figure 8:**
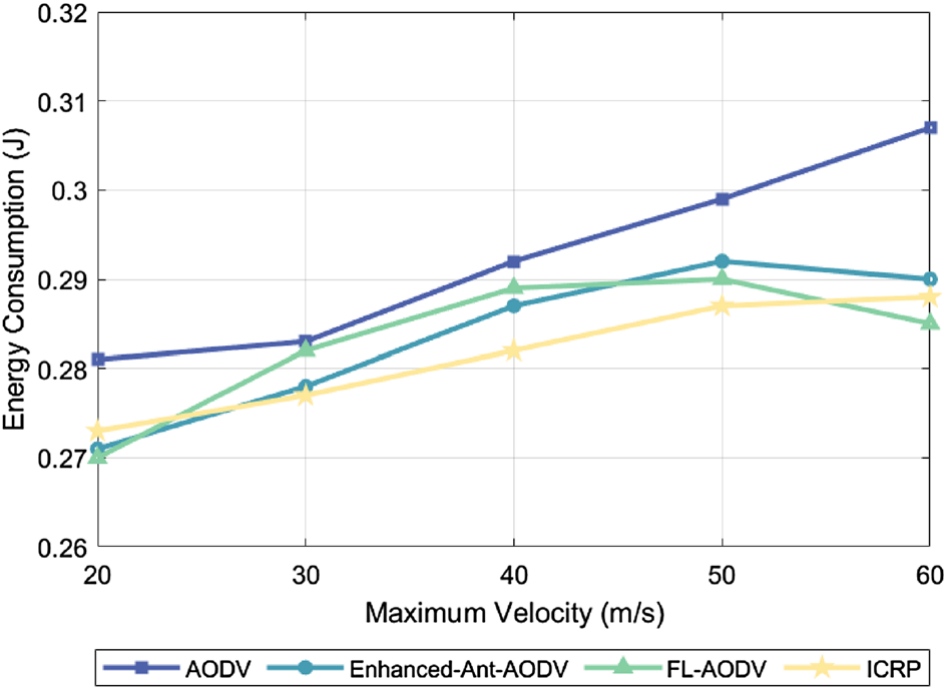
Performance comparison of energy consumption at different maximum node speeds.

In a resource-constrained FANET environment, if any drone exits due to energy depletion, the network’s connectivity and stability will be significantly affected. Low energy consumption can reduce node failures due to insufficient power, maintaining network integrity and efficient communication. For routing protocols, intelligent selection of the optimal transmission path significantly reduces communication energy consumption, data retransmission, and latency, while adapting to dynamic network changes, ensuring the continuity of the optimal transmission path. This strategy not only extends the device’s lifespan but also enhances network stability, which is crucial for improving overall network efficiency and supporting long-term operation. Figure 7 shows the relationship between energy consumption corresponding to four different routing protocols and the simulated number of drones under the condition of a maximum flight speed of 60 m/s. Figure 8 illustrates the comparison of the energy consumption of 100 drones using these four routing protocols at different maximum flight speeds. Both figures indicate that the energy consumption increases as the number of drones or the flight spead increases. This trend can be attributed to the increase in the number of UAVs or their flight speed complicating the topology of the flight self-organizing network. This complexity in topology increases the control overhead, subsequently leading to an increase in energy consumption. ICRP, as proposed in this paper, exhibited relatively stable energy consumption compared to the Enhanced-Ant-AODV and FL-AODV protocols. When compared to the AODV routing protocol, ICRP showed a decrease in energy consumption by 4.28% and 3.72%, respectively. The performance improvement is attributed not only to ICRP considering path length when establishing routes but also to emphasizing path stability. Additionally, the protocol introduces predictive repair and contraction mechanisms after route establishment, enabling dynamic maintenance of routes. Although this increases control overhead, it helps reduce packet retransmissions and detour phenomena, making the ICRP routing protocol more adaptive in dynamically changing scenarios compared to others, with a more gradual increase in energy consumption.

### Routing establishment time

**Figure 9:**
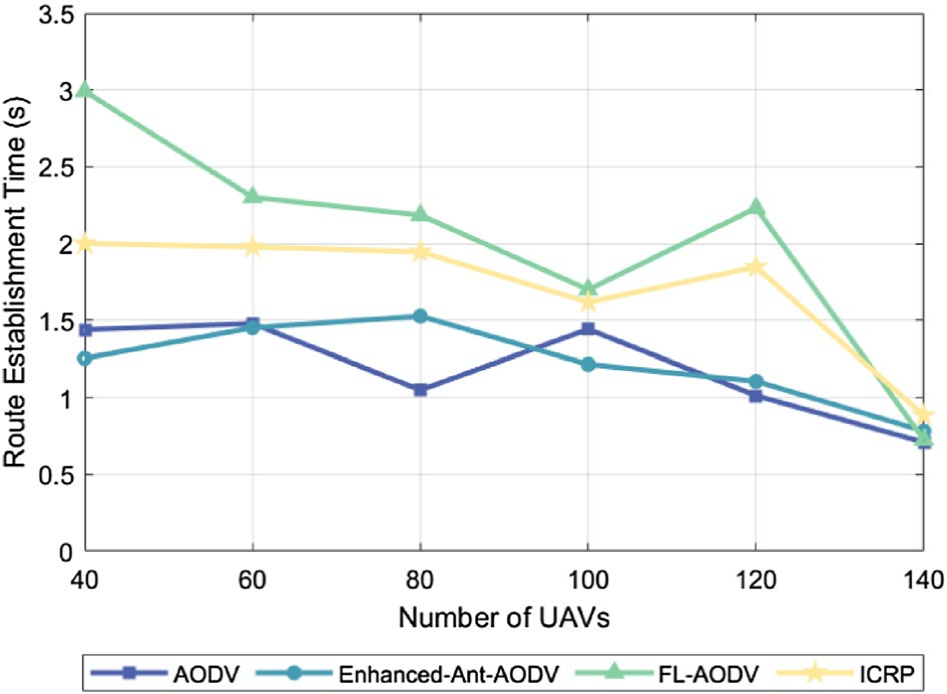
Performance comparison of routing establishment time with different number of nodes.

**Figure 10:**
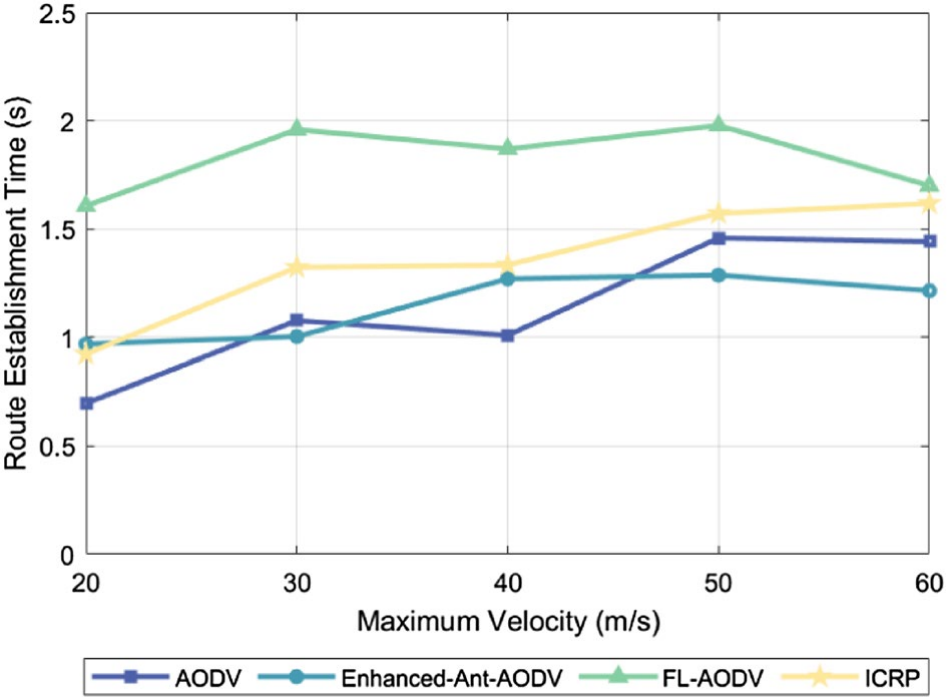
Performance comparison of routing establishment time at different maximum node speeds.

The routing establishment time measures the overall duration from the moment a source node initiates a routing request to the successful establishment of a routing connection. This metric can reflect the complexity of a protocol, since more complex protocols tend to involve more data exchange, calculations, and confirmation steps to ensure effective routing establishment. Thus, the more complex operations and procedures included in a protocol, the longer the routing establishment time. Figure 9 illustrates the relationship between the routing establishment time of four different routing protocols and the number of simulated drones under the condition of a maximum flight speed of 60 m/s. The observation results show that the routing establishment time exhibits a downward trend as the number of drones increases. This phenomenon can be attributed to the clustering network structure adopted by these four routing protocols, effectively limiting the surge in control packet exchanges caused by the increase in node count, while enhancing the likelihood of establishing routing and reducing network fragmentation. Figure 10 presents the routing establishment time of these four routing protocols when 100 drones are utilized under varying maximum flight speeds. The results indicate that the routing establishment time increases as the flight speed increases. This is because higher flight speeds intensify the dynamic nature of the network topology, requiring routing protocols to devote more time to adapt to and calculating the optimal routing paths. Among the four protocols shown in these two figures, AODV and Enhanced-Ant-AODV exhibit relatively shorter routing establishment times, followed by the ICRP proposed in this study, and FL-AODV having the longest routing establishment time. The reason for this discrepancy lies in the fact that AODV and Enhanced-Ant-AODV only involve backbone network nodes in sending control packets during the routing establishment process, whether it is searching for destination nodes or establishing reverse routing, resulting in relatively lower computational complexity. By contrast, the ICRP protocol, during the forward search for destination nodes, not only requires backbone network nodes to forward control packets, but also demands other nodes to broadcast their pheromone concentrations to neighboring nodes, consuming additional channel resources and increasing time overhead. Furthermore, during the reverse routing establishment, the number of candidate nodes increases to avoid using key backbone network nodes as relay nodes, further augmenting computational complexity and the routing establishment time. In the FL-AODV protocol, upon receiving routing request packets during the forward search for destination nodes, it does not immediately forward them. Rather, it collects multiple routing requests, selects a relatively stable link as the reverse routing path, and forwards the routing requests. While this strategy improves the stability of the established routing, it is not suitable for highly dynamic scenarios, resulting in longer routing establishment times and even potential failures in establishing routing.

## Conclusion

To address the problems of excessive burden on cluster-head nodes and poor adaptability to highly dynamic topologies in existing inter-cluster routing protocols, this study proposed an inter-cluster routing protocol based on a hybrid ant colony algorithm. During the route establishment phase, the node pheromone is calculated by comprehensively considering the number of hops from the source node, node load, and node weights. Additionally, the foraging behavior of Physarum polycephalum is integrated as a heuristic function to evaluate the stability of links between nodes. This approach optimizes relay node selection and enhances routing stability. In the route maintenance phase, the protocol employs predictive repair and contraction mechanisms to dynamically adjust established routes. This adjustment reduces route disconnections and the number of redundant relay nodes, further improving link stability. Experimental results showed that our proposed routing protocol performs better in stability and reliability than the existing routing protocols. However, the potential issue of information leakage inherent in wireless communication has not been addressed in this study. Future research will focus on enhancing the secure transmission of routing information while ensuring the stability of network routing links. In the future, we plan to further explore and integrate advanced encryption technologies suitable for wireless network environments, such as quantum encryption and AES encryption, and authentication mechanisms, such as certificate-based PKI systems, to ensure the confidentiality and integrity of data transmission. Additionally, we will focus on the development of future network technologies, including 5G/6G and IoT, to ensure that our protocols can adapt to new network technology frameworks, support cross-platform and cross-technology interoperability, and lay the foundation for building a more secure, stable, and widely applicable network communication environment.

## Data Availability

All data generated or analyzed during this study are included in this published article.
